# Zinc in Regulating Protein Kinases and Phosphatases in Neurodegenerative Diseases

**DOI:** 10.3390/biom12060785

**Published:** 2022-06-04

**Authors:** Hui-Liang Zhang, Xiao-Chuan Wang, Rong Liu

**Affiliations:** Department of Pathophysiology, Key Laboratory of Ministry of Education for Neurological Disorders, School of Basic Medicine, Tongji Medical College, Huazhong University of Science and Technology, Wuhan 430030, China; d202181578@hust.edu.cn (H.-L.Z.); wxch@mails.tjmu.edu.cn (X.-C.W.)

**Keywords:** zinc, protein kinases, protein phosphatases, neurodegenerative diseases, Alzheimer’s disease

## Abstract

Zinc is essential for human growth and development. As a trace nutrient, zinc plays important roles in numerous signal transduction pathways involved in distinct physiologic or pathologic processes. Protein phosphorylation is a posttranslational modification which regulates protein activity, degradation, and interaction with other molecules. Protein kinases (PKs) and phosphatases (PPs), with their effects of adding phosphate to or removing phosphate from certain substrates, are master regulators in controlling the phosphorylation of proteins. In this review, we summarize the disturbance of zinc homeostasis and role of zinc disturbance in regulating protein kinases and protein phosphatases in neurodegenerative diseases, with the focus of that in Alzheimer’s disease, providing a new perspective for understanding the mechanisms of these neurologic diseases.

## 1. Introduction

### 1.1. Zinc Balance in the Human Body

**Figure 1:**
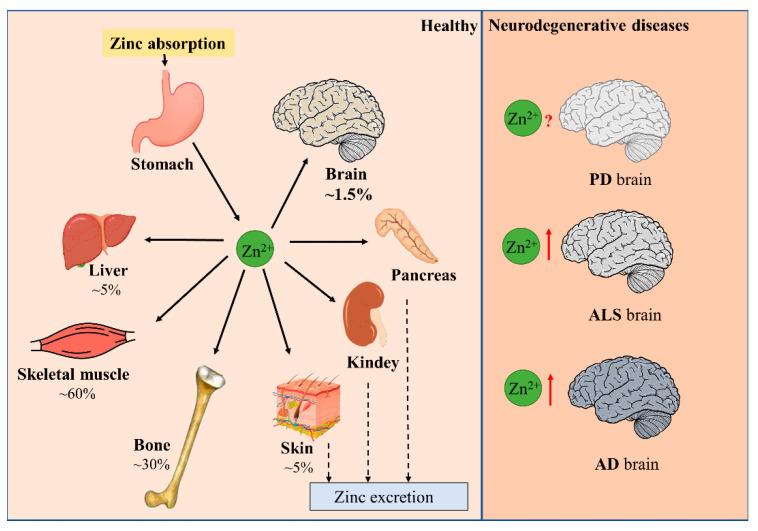
Distribution of zinc in the body. Dietary zinc is absorbed by the digestive system and then distributed to peripheral tissues. Approximately 60% of zinc is stored in skeletal muscle, 30% in bone, and 5% in liver and skin. Excess zinc is excreted through gastrointestinal secretions and mucosal cells. Brain zinc levels change in patients with neurodegenerative diseases such as PD, ALS, and AD.

Minerals in the human body that are less than 0.01% of body weight are called micronutrients. Zinc is an indispensable trace nutrient for life. Initially, zinc ions were only found to be essential for the normal growth and development of plants and animals [1,2], and it was not until 1961 that zinc was identified as an essential micronutrient for human beings. It is currently ranked first among the 14 essential micronutrients identified by the World Health Organization. Zinc is also the second most abundant micronutrient in the human body, following only iron. The adult human body contains about 2–3 g of zinc, of which 60% is found in skeletal muscle, 30% in bone, 5% in liver and skin, 1.5% in brain, and the kidneys, heart, and other tissues in total contribute less than 2% [3]. Serum zinc occupies only 0.1% of total body zinc, which is exactly the daily level of zinc intake. Zinc homeostasis is tightly controlled through the coordinated regulation of zinc absorption, efflux, distribution, and storage (Figure 1) [4,5]. Zinc performs unique and broad functions in numerous biological processes, including cell division, growth, differentiation, and signal transduction. In these processes, zinc ions serve as structural, catalytic, or regulatory components in proteins, such as transcription factors, enzymes, transporter proteins, and receptors [5,6,7]. Hence, zinc is extremely valuable to the organism. Disruption in zinc homeostasis is found to be associated with a variety of diseases, including asthma, diabetes, and Alzheimer’s disease [8,9,10].

### 1.2. Distribution of Zinc and the Maintenance of Zinc Homeostasis in the Brain 

The adult human brain contains 0.04 g of zinc, accounting for only 1.5% of the total body zinc [3]. There are three zinc pools in the brain: vesicular zinc, protein-bound zinc, and free zinc ions. Ultrastructural observation shows that abundant free zinc ions are present in presynaptic glutamatergic vesicles called “zincergic” synapses. Vesicular zinc is found in the cerebral cortex, amygdala, hippocampus, and olfactory bulb, which accounts for 5~15% of the brain’s zinc content and is especially abundant in the hippocampus [11,12]. Endogenous zinc homeostasis is mainly regulated by zinc-binding proteins, metallothioneins (MTs), and zinc transporter proteins. There are four MTs in human and mammalian cells, of which MT-3 is dominated in the brain. MT-3 serves to bind and store zinc and thus protect cells from the toxic effects of excessive free zinc ions [13]. Zinc transport proteins are divided into two families: the ZnTs (zinc transporter proteins) and the ZIPs (Zrt and Trt-like proteins). ZnTs are responsible for removing zinc out of the cells or transporting zinc into cellular organelles, thus reducing the concentration of zinc in the cytoplasm, while ZIPs play the opposite role [14,15]. There is a growing body of evidence showing that ZnTs and ZIPs play an instrumental role in maintaining zinc homeostasis, which profoundly affects human health, directly or indirectly [16].

Zinc is required for normal brain development, and it also plays important roles in the physiological function of the central nervous system (CNS) [17]. The average intracellular zinc ion concentration in brain cells is about 150 μM [18,19]. Zinc ion concentration in the synaptic cleft is 15–30 μM under the resting state and can be up to about 300 μM during neural activity [20,21]. Sub-toxic doses of zinc can cause the activation of microglia (>30 μM) or astrocytes (>100 μM) via the NF-κB or Stat3 pathways, respectively [22,23]; higher concentrations of exogenously applied zinc (>300 μM) can cause neuronal death in vitro [24]. The increase in intracellular zinc ions caused by noxious stimuli can also lead to extensive neuronal death [25]. Owing to the multifaceted roles of zinc in biological processes, zinc dyshomeostasis in the brain participates in the development of many neurodegenerative diseases [26].

### 1.3. Limited Effect of Peripheral Zinc Supplementation on Brain Zinc Level

Zinc homeostasis in the brain is tightly controlled and is not easily altered by a peripheral zinc load. It was shown that a slight increase in ZnT2 was observed after treatment of the BBB model with “moderate zinc excess” (50 µM), with no significant difference in the expression of ZIP1. Our previous investigation also showed that high-dose zinc supplementation significantly increased serum zinc levels in mice but did not increase brain zinc levels. On the contrary, decreased zinc levels were observed in the hippocampal brain region, which may be a result of the loss of hippocampal neurons (abundant with zinc) induced by the toxicity of excessive zinc supplementation [27]. 

## 2. Disturbance of Zinc Homeostasis in Neurodegenerative Diseases 

Due to the multiple roles of zinc in lots of physiological processes, the maintenance of zinc homeostasis is of particular importance for normal brain function. Either zinc deficiency or overload may be harmful and may exacerbate brain damage in neurological disorders [26]. In addition, even though the total zinc in the brain is normal, the mislocalization of zinc ions, for example, abnormal zinc accumulation with pathologic proteins, or excessive zinc release from intracellular zinc-binding proteins may also result in dysfunction of the nervous system. The important role of zinc in the brain has led to the exploration of the effects of zinc disturbance in many neurodegenerative diseases, particularly in Parkinson’s disease (PD), amyotrophic lateral sclerosis (ALS), and Alzheimer’s disease (AD) [28,29,30,31]. 

### 2.1. Parkinson’s Disease (PD)

Parkinson’s disease (PD) is a severe motor disorder characterized by tonicity, tremor, and bradykinesia. In the PD brain, α-synuclein aggregates, and neurofibrillary tangles are formed in the substantia nigra pars compacta, with a selective loss of dopaminergic neurons [32]. The deficit of dopaminergic neurons in substantia nigra causes a striatal dopamine deficiency and subsequent alteration in basal ganglia physiology, which is thought to be the key mechanism leading to the development of clinical symptoms [33,34]. Dopamine neurons in the ventral tegmental area (VTA), which is adjacent to the substantia nigra and regulates emotional behavior, are less damaged [35,36].

The relationship between zinc dyshomeostasis and PD is ambiguous, with some studies showing reduced serum zinc levels [37,38], with others showing increased serum zinc levels in PD patients. No significant change in cerebrospinal fluid zinc levels was observed [39,40]. Evidence supporting the relationship between low zinc and PD is that methamphetamine causes dopaminergic cell death through the production of reactive oxygen species and increasing the total amount of α-synuclein (α-syn), a major component of Lewy bodies, and zinc pretreatment reverses the above phenomena by increasing metallothionein expression in vitro [41,42]. In the Drosophila PD model, zinc supplementation also increased the lifespan and motility [43]. On the other hand, there is numerous evidence showing the toxicity of excess zinc ions in PD-related degenerated dopaminergic neurons. Zinc exposure is an environmental risk factor for PD [44]. The autopsy results showed zinc deposition in the substantia nigra and striatum in PD patients [45]. Consistent with these findings, zinc treatment significantly enhanced MPTP-induced dopaminergic neuronal loss in mice [34,46]. Taken together, these studies suggest that the imbalance of zinc may play a dual role in the development of PD, which depends on the different signaling pathways influenced by zinc in different disease stages.

### 2.2. Amyotrophic Lateral Sclerosis (ALS)

Amyotrophic lateral sclerosis (ALS) is an incurable neurodegenerative disease in which motor neurons in the spinal cord and brain progressively deteriorate, leading to progressive muscle atrophy, paralysis, and ultimately death. The most commonly studied risk factors for ALS include genetics, environment, and lifestyle. Metals with a potential relationship with ALS progress include copper, aluminum, arsenic, cadmium, cobalt, zinc, vanadium, and uranium, all of which are significantly elevated in the cerebrospinal fluid of ALS patients [47], while the precise roles of these metals in the brain in disease progression need further exploration. 

It is suspected that aggregates of a protein called copper–zinc superoxide dismutase 1 (SOD1) in nerve cells may play a crucial role in disease development. More than 140 SOD1 gene mutations have been found associated with ALS, with varying degrees of severity. TDP-43 gene mutation is also found in 5–10% of familial ALS cases. Significantly increased levels of Mn, Cu, and Zn were found in the spinal cord of TDP-43 A315T mice [48]. The immunoreactive activity of metallothionein was found to be elevated in the brain and liver in ALS patients [49]. Similarly, in SOD1-Gly93Ala transgenic mice, increased MT1, MT2, and MT3 expression in astrocytes and increased MT3 in neurons were observed. The elevated expression of metallothionein may be compensatory. Taking together, it seems that zinc exerts deleterious effects during ALS development. 

### 2.3. Alzheimer’s Disease (AD)

Alzheimer’s disease (AD) is a progressive neurodegeneration that is currently receiving the most attention in aging-related disorders. It is expected that by 2050, there will be 130 million AD patients worldwide [50,51]. AD patients exhibit progressive cognitive decline manifested by memory loss, learning and orientation difficulties, behavioral changes, and dementia [51]. Amyloid plaques aggregated by β-amyloid (Aβ) peptides and neuronal fibrillary tangles (NFTs) formed by hyperphosphorylated tau protein are two typical pathological features in AD brain [52,53]. Zinc dyshomeostasis was one of the dominated pathological characters in AD. The levels of zinc ions in the amygdala, hippocampus, cerebellum, and superior temporal gyrus, which are all susceptible brain regions in AD, were found elevated [54,55,56]. These changes may be a result of the capture of zinc by Aβ. Zinc was highly enriched in senile plaques in the brains of AD mice [57]. Amyloid precursor protein (APP) and Aβ have zinc-binding sites [58], and studies show that zinc promotes Aβ aggregation directly in vitro [59,60]. On the other side, zinc exacerbated Aβ toxicity, and zinc chelation into amyloid deposits led to the loss of functional zinc at the synapse [61]. Besides zinc ions, zinc transporters also showed significant changes in AD brain. The levels of ZIPs, ZnTs, and MTs, which regulate brain zinc levels, were increased or decreased in the early or late stages of AD [62,63,64]. Postmortem studies of AD brains showed a significant decrease in ZnT-1, along with an increase in ZnT-6 in the hippocampus/parahippocampal gyrus compared to normal control subjects. These processes correlate with disease progression and the severity of cognitive impairment [64,65]. The expression of ZnTs (ZnT1, ZnT3, ZnT4, ZnT6, and ZnT7) were extensively increased in the senile plaque of APP/PS1 mouse models [16,58]. Till now, what is the initial cause of zinc disturbance, and whether the changes of zinc transporters are causes or results of zinc disturbance in AD, remain unclarified.

The pathologic role of zinc in AD is also manifested by the effects of zinc on promoting tauopathy [61,66]. Zinc can cause tau hyperphosphorylation through inhibiting the protein-phosphatase 2A (PP-2A) [67] and activating tau kinases [68,69]. All of these mechanisms lead to increased Aβ deposition and P-tau, ultimately exacerbating the pathological progression of AD.

## 3. Zinc-Regulated Protein Kinases and Protein Phosphatases

**Figure 2:**
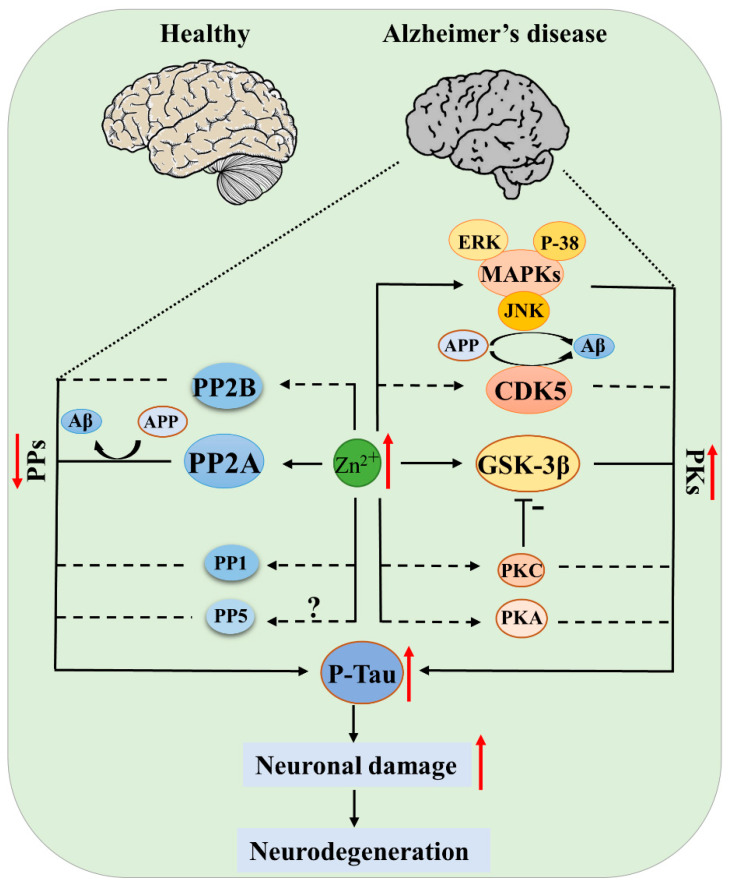
Protein kinases (PKs) and protein phosphatases (PPs) of tau regulated by zinc in AD. Elevated zinc causes activation of kinases (GSK-3β, CDK5, MAPK, PKA, PKC) and simultaneous inhibition of phosphatases (PP-2A, PP-2B), leading to tau pathogenesis; the inhibition of PP-2A and activation of JNK, CDK5 also promote APP hyperphosphorylation and Aβ deposition, ultimately resulting in neuronal damage and disease development. Solid lines indicate there is known evidence from literatures, and dashed lines indicate that direct evidence is still absent, but current data support the hypothesis.

Due to the multiple functions of zinc in cell growth and differentiation, metabolism, apoptosis, and neurogenesis, zinc disturbance may participate in the development of neurodegenerative diseases through distinct molecular mechanisms in different cell types and disease stages. Especially in the development of AD, these mechanisms have been thoroughly discussed in several outstanding reviews [70,71,72]. Protein phosphorylation is a posttranslational modification that regulates the protein activity, degradation, and interaction with other molecules. Protein kinases (PKs) and phosphatases (PPs), with their effects of adding phosphate to or removing phosphate from the substrates, are key regulators in controlling the phosphorylation of proteins. Abnormal protein phosphorylation is a common pathologic event in neurodegenerative diseases. For example, APP and tau were hyperphosphorylated in the AD brain, and hyperphosphorylated α-synuclein was found in the PD brain, while for ALS, it was TDP-43 that was hyperphosphorylated [70,72]. In this review, we discuss the role of zinc in regulating protein phosphorylation by influencing the structures or activities of protein kinases and protein phosphatases, with the focus on tau kinases and phosphatases (Figure 2). 

### 3.1. Zinc and Protein Kinases

Kinases that phosphorylate tau are classified into two types: proline-directed kinases, such as glycogen synthase kinase 3β (GSK-3β), cyclin-dependent kinase 5 (CDK5), MAP kinase (p38, ERK, c-JNK), and other stress kinases; and non-proline-directed kinases, such as protein kinase A (PKA), protein kinase C (PKC), calmodulin (CaM) kinase II, and MARK kinase [73,74,75,76,77].

#### 3.1.1. Proline-Directed Kinases

##### GSK-3β

Glycogen synthase kinase-3beta (GSK-3β) is an evolutionarily conserved serine/threonine kinase that was first discovered for its role in glycogen synthesis [78]. GSK-3β exerts its function in numerous cellular processes including cell proliferation, DNA repair, cell cycle, and metabolic signaling pathways. So far, GSK-3β is considered one of the most favorable tau kinases [79,80]. Zinc ions can activate GSK-3β. Treatment of SY5Y neuroblastoma cells with 100 μM zinc sulfate induced GSK-3β phosphorylation at Ser9 and Tyr216, which were reversed by PI3K and MEK1/2 inhibitors respectively, together with increased phosphorylation of tau [81]. It is known that GSK-3β phosphorylation at Tyr216 activates GSK-3β, whereas Ser9 phosphorylation results in GSK-3β inhibition [82]. Although the activity of GSK-3β was not measured in this study, another experiment indicated that in cultured hippocampal progenitor H19-7 cells, upon exposure to zinc (10 μM) and zinc-specific ionophore pyrithione, GSK-3β was phosphorylated at Ser9 and tyrosine sites (not identified), and enzyme activity assay indicated the activation of GSK-3β. At the same time, tau was hyperphosphorylated. Inhibition of PI3K/Akt or overexpression of kinase-deficient Akt (KD-Akt) rescued these changes induced by zinc; tau phosphorylation was reversed by the overexpression of kinase-deficient GSK-3β or its dominant-negative mutant [83]. These data collectively indicate that zinc can activate GSK-3β, thus promoting tau phosphorylation. However, the precise molecular mechanism of GSK-3β activation by zinc needs further clarification. For example, tyrosine kinases/phosphatases contributing to GSK-3β Tyr216 phosphorylation in zinc treatment have not been identified. 

In addition to regulating tau phosphorylation, GSK-3β increased BACE1 gene expression through its effect on the BACE1 promoter, which in turn promoted Aβ production, whereas the administration of ARA (a GSK inhibitor) decreased BACE1-mediated APP processing and the production of C99 and Aβ in vitro [84]. The ability of GSK-3β to contribute to the phosphorylation of tau and Aβ production provides a link between the two pathologies and suggests a key role for GSK-3β in the pathogenesis of Alzheimer’s disease, where zinc ions may be involved through activating GSK-3β.

##### CDK5

Cyclin-dependent kinase 5 (CDK5) is a member of the cyclin-dependent kinases (CDKs), and its full activity in cells requires the binding of p35 or p25 regulatory subunits, which are highly enriched in neurons. Different groups have identified the phosphorylation sites of CDK5 on tau using amino acid sequencing or mass spectrometry, with Ser202, Thr205, Ser235, and Ser404 being the most favorable [85,86]. Zinc can induce CDK5 activation through phosphorylation of CDK5 at Tyr15 in rat brain with ischemic injury [87]. In addition, it is known that iron ions can induce the phosphorylation of tau via CDK5 in the mouse hippocampus, while it remains to be explored whether zinc ions can exert a similar effect [88]. APP can also be phosphorylated at Thr668 by CDK5 in neuronal cultures in vitro [89], suggesting an important role of CDK5 in promoting amyloidosis in AD. Based on these findings, it is reasonable to speculate that tau and APP phosphorylation by CDK5 may be induced by zinc, which needs further identification.

##### MAPKs (p38, ERK, JNK)

MAPKs are serine/threonine protein kinases that regulate the cellular signals in response to a wide range of extracellular stimuli and play important roles in the regulation of cell proliferation, differentiation, survival, and apoptosis. The mitogen-activated protein kinase (MAPK) cascade reaction consists of three core kinases: MKKK, MKK and MAPK. The top kinase of the triple kinase module is MAPK kinase kinase (MKKK). MKKK is a serine/threonine kinase that, when activated, phosphorylates the next kinase in the module, MAPK kinase (MKK). In turn, these kinases activate MAPK by directly phosphorylating the Tyr and Thr residues in their activation loop motifs. MAPK is the final kinase in the triple kinase module, phosphorylating substrates on serine and threonine residues [90]. In mammalian cells, several distinct MAPKs have been identified, mainly including p38 MAPK, c-jun N-terminal kinase (JNK), and extracellular signal-regulated kinase (ERK 1/2). Multiple studies have shown the activation of MAPKs in AD brains and their important roles in mediating tau phosphorylation in cell and animal models [91]. 

Zinc may regulate the activity of MAPKs directly or indirectly through regulating the upstream MKKKs or MKKs. Most evidence is from cells treated by extracellular zinc incubation or inducible intracellular zinc release, and evidence for the direct regulation of zinc on enzyme activities of MAPKs is lacking. DTDP-induced intracellular zinc release was found to activate p38 and cause neuronal death in vitro [92]. Elevated zinc ions can activate ERK through the MAPK cascade of Ras/Raf/MEK and eventually lead to differentiated PC12 cells death. The researchers also observed the activation of JNK, but the signal pathway downstream of zinc leading to JNK activation was not further explored since JNK activation seemed to not participate in cell death by zinc in this experiment [93]. In the presence of 100 μM zinc ions, astrocytes can be activated through the ERK/Stat3 pathway in vitro [22]. 

In relating to tau pathology, zinc ions can activate ERK via the Ras/Raf/MEK pathway, ultimately causing tau S214 phosphorylation in human wild-type SH-SY5Y cells expressing tau_1-441_ [69]. A study on APP phosphorylation showed that JNK also plays an important role in APP metabolism, as the JNK inhibitory peptide (D-JNKI1) prevents APP phosphorylation at Thr668, leading to a significant decrease in βAPP and Aβ levels in H4-APP cell line [94]. Administration of SP600125, a JNK-specific inhibitor, significantly reversed amyloid plaque burden and synaptic loss in APP/PS1 transgenic mice [95]. Given the role of MAPKs in APP and tau phosphorylation, several inhibitors targeting ERK, JNK or p38 have been developed as potential drugs for AD therapy [95,96,97]. 

#### 3.1.2. Non-Proline-Directed Kinases

##### PKA

Protein kinase A (PKA) is a tetramer composed of two regulatory protein subunits, R, and two catalytic subunits, C. When the second messenger cAMP binds to the R subunit, it causes PKA activation. The phosphorylation of tau at Ser214 and PKA activity are increased simultaneously in the dorsolateral prefrontal association cortex of macaques, suggesting that tau phosphorylation may result from PKA activation. Moreover, the activation of PKA by forskolin (Fsk) produced a dose-dependent increase in the phosphorylation of tau at Ser214 in mouse primary cortical neurons [98,99]. In a study on sperm motility, extracellular zinc was identified to bind and activate the Zn^2+^ sensing receptor, which further activates adenylyl-cyclase (AC) to increase [cAMP]i and activate PKA [100]. Another experiment also showed that zinc exposure caused the activation of the cAMP/PKA pathway in human bone marrow-derived mesenchymal stem cells [101]. However, till now there is no direct evidence supporting that zinc induces the phosphorylation of tau via the cAMP/PKA pathway.

##### PKC

Protein kinase C (PKC), consisting of 12 isozymes, is divided into three subfamilies (classic PKC, novel PKC, and atypical PKC). The second messenger DAG and Ca^2+^ play a master role in PKC activation [102,103,104]. PKC not only induces the phosphorylation of tau at the Thr231 and Ser313 sites, but also the phosphorylation of GSK3β at Ser-9, thereby inhibiting GSK3β activity as well as GSK3β-induced phosphorylation of tau at Ser202, Thr205, and Thr181 in vitro [105,106,107]. These results suggest a complex effect of PKC on GSK3β and tau. In neuronal cultures, zinc induces the activation of PKC, which activates NADPH oxidase, accompanied by the production of nitric oxide synthase (NOS) [108]. Short-term exposure to 300 μM zinc caused increased PKC activity and cortical neuronal death in vitro, which were eliminated by Ca-EDTA or PKC inhibitors. This suggests that PKC also plays an important role in zinc-induced neuronal death [109]. Insulin-induced increases in Aβ_40/42_ and phosphorylation of tau at Thr231 were blocked by a PKC inhibitor ACPD in both cultured neuronal cells and mouse hippocampal slices [110]. The above results suggest that PKC is also a potential kinase mediating zinc-induced tau/APP phosphorylation in AD. 

### 3.2. Zinc and Protein Phosphatases

Proteomic analysis of 6600 phosphorylation sites on thousands of human proteins showed that phosphoserine (pSer), phosphothreonine (pThr) and phosphotyrosine (pTyr) accounted for 86.4%, 11.8% and 1.8% of phosphorylated amino acids, respectively [111]. Protein serine/threonine phosphatases (PSPs), which are responsible for the removal of phosphate from target proteins on serine and threonine sites, participate in many cellular functions, such as cell cycle, growth, metabolism, transformation, and apoptosis [6]. PSPs are classified as three families: phosphoprotein phosphatases (PPP), metal-dependent protein phosphatases (PPM), and aspartic acid-based phosphatases (FCP/SCP, TFIIF-associating component of RNA polymerase II CTD phosphatase/small CTD phosphatase). The general classification of such phosphatases is based on the primary structure and catalytic mechanism of the enzyme. The PPM family are metal-dependent protein phosphatases that act as single-subunit enzymes bound to Mn^2+^ or Mg^2+^. This family is also known as the PP2C family as members of PPP. It has been reported that the dysregulation of PPM phosphatases may be associated with the development of tumorigenesis, metabolic disorders, and cancers. Currently, its role in neurodegenerative diseases is under determination [112]; FCP/SCP uses an aspartic acid-based catalytic mechanism, and its only known substrate is the C-terminal structural domain (CTD) of RNA polymerase II, which contains a tandem repeat sequence of a serine-rich heptapeptide [113]. Among three PSPs, zinc ions are rarely reported with PPM or FCP/SCP; here, we mainly focus on PPP, which can be further classified into three types: PP-1, PP-2 (PP-2A, PP-2B), and PP-5. 

#### 3.2.1. PP-1

Protein phosphatase 1 is a ubiquitously expressed serine/threonine phosphatase. In AD brain, the activity of PP-1 is decreased by ~20% [114]. In a study comparing the dephosphorylation ability toward tau of different tau phosphatases, PP-1 was found to account for ~11% of total brain tau phosphatase activity [115]. However, autopsy of AD brain revealed that phosphorylated tau at Thr212 could only be dephosphorylated by purified PP-1 but not by PP-2A/PP-2B [116]. PP-1 can be inhibited by many toxins, such as OA and tautomycin, as well as endogenous protein inhibitors [113,117]. For the recombinant catalytic subunit of PP-1, its activation requires the addition of Mn^2+^ [118]. Chu et al. further investigated the effects of other divalent cations on PP-1 activity and found that treatment of the recombinant PP-1 catalytic subunit with a combination of Fe^2+^ and Zn^2+^ (but not the individual metal ions) significantly activated the phosphatase. These results suggest that PP-1 may be an iron/zinc metalloprotein in vivo [119]. Till now, there are still no studies that investigated the effect of elevated extracellular zinc on PP-1 activity in cells. In a study exploring the toxicity of aluminum in rats, Al treatment increased the protein level of GSK-3, whereas it decreased the protein level of PP-1α in the brain, which were reversed upon zinc supplementation in drinking water. However, the underlying mechanisms were not further explored [120].

#### 3.2.2. PP-2A

Protein phosphatase 2A (PP-2A) is the major serine/threonine phosphatase in eukaryotic cells, which contributes to ~71% of total brain tau phosphatase activity [115]. In the AD brain, the activity of PP-1, PP-2A, and PP-5 are decreased [114,121]. PP-2A inhibition can cause the hyperphosphorylation of tau at multiple AD-related phosphorylation sites [122], as well as APP phosphorylation at Thr668 [123]. Both tau hyperphosphorylation and Aβ deposition are typical pathological features of AD [124,125]. Just as PP-1, the activity of PP-2A catalytic subunit is also regulated by ions. A study compared the activity of the core enzyme of PP-2A, which is composed of a catalytic C subunit and a scaffolding A subunit, under different ion concentrations in vitro [126]. In the mammalian brain, the major core enzyme of PP-2A is CA [127,128]. The researchers reported that the Mn^2+^-dependent C′A’ and -independent CA PP2A can be purified from human erythrocytes, and CA is a Zn^2+^- and Fe^2+^-metalloenzyme. For the Mn^2+^-independent PP-2A (C’A’), ZnCl_2_ at a concentration lower than 10 μM stimulated the phosphatase activity, whereas at higher concentrations, this effect was attenuated. The PP and PTP activities of CA were unaffected by the same zinc and/or iron treatment [126]. We explored the direct effect of zinc on PP-2A activity through incubating neuronal cell lysates or purified recombinant PP-2A catalytic subunit with zinc in vitro, and found that zinc at a concentration of 10 μM inhibited PP-2A directly, and the binding site located in segment PP2Ac (51–270) [129]. Thus, whether excessive zinc inhibits PP-2A in the brain, depends on the subunit composition of PP-2A and the exact zinc concentration. While zinc concentration may exceed 10 μM under lots of pathologic conditions, such as during ischemia, or in zinc-accumulated senile plaques in AD, it is reasonable to speculate a direct inhibitory effect of zinc on PP-2A exists in the development of brain disorders, such as stroke and AD. 

In the brain, excessive zinc may also regulate the PP-2A activity indirectly through cell signaling transduction. Y307 phosphorylation of PP-2A catalytic subunit was found to potently inhibit PP-2A activity [130]. In cultured rat hippocampal slices, synaptic released zinc promotes tau hyperphosphorylation through inhibiting PP-2A [67]. In cells and the rat brain, zinc induced PP-2A inactivation and tau hyperphosphorylation through Src-dependent PP2A-Y307 phosphorylation [67,131]. These findings suggest that zinc disturbance is an important upstream factor contributing to PP-2A inhibition in AD.

#### 3.2.3. Calcineurin/PP-2B

Calcineurin is a calcium- and calmodulin-dependent enzyme found in many cell types, especially enriched in neuronal soma and processes. Calcineurin comprises a catalytic “A” subunit, which interacts with calmodulin in a calcium-dependent manner, and a regulatory “B” subunit [132]. Calcineurin can dephosphorylate tau at a few sites, but it accounts for only ~7% of total brain tau phosphatase activity [115,133,134]. Decreased expression of calcineurin contributes to abnormal tau hyperphosphorylation in mouse models of Huntington’s disease, an autosomal dominant neurodegenerative disorder [135]. Calcineurin contains Zn^2+^ in its catalytic domain and its activity can also be directly regulated by exogenous zinc. It was reported that Zn^2+^ at a concentration of 10 nM–10 μM inhibited Ni^2+^-stimulated-calcineurin activity in a dose-dependent manner in vitro [136]. The inhibitory effect of zinc on calcineurin was also observed in cell experiments. Zinc at 30–100 μM inhibited the osteoclastogesis induced by receptor activator of NF-κB ligand (RANKL) in mouse bone marrow-derived monocytes. During this process, the calcineurin activity decreased in response to zinc but its protein level was unchanged. A similar result was observed in doxorubicin (DOX)-treated H9c2 rat cardiac muscle cells: pretreatment of H9c2 cells with exogenous zinc attenuated the DOX-activated calcineurin signaling in a dose-dependent manner [137,138]. These findings collectively suggest an inhibitory effect of zinc on calcineurin. In neurological-related diseases, this inhibition may promote the hyperphosphorylation of calcineurin substrates, such as tau protein.

#### 3.2.4. PP-5

Protein phosphatase 5 is ubiquitously expressed in all mammalian tissues, with a high level in the brain. In the presence of arachidonic acid, PP-5 dephosphorylates tau at many of the sites that are hyperphosphorylated in AD in vitro [139]. The activity, but not the protein level of PP5, was found to be decreased by approximately 20% in AD neocortex [114,121]. Since PP-5 is a late-identified phosphatase, its interaction with zinc remains to be explored. 

A summary of the changes of zinc-regulated kinases/phosphatases in neurodegenerative diseases, and the possible regulating mechanisms of zinc, was list in Table 1. 

## 4. Conclusions

Zinc is one of the most important micronutrients. Zinc homeostasis is of great importance for the central nervous system. Disturbed zinc metabolism is prominent in neurodegenerative disorders, such as PD, ALS, and AD, which may participate in the disease progress in multiple pathways. The abnormal hyperphosphorylation of proteins, such as tau and APP, is a hallmark in these diseases. Zinc can regulate the activity of related protein kinases and phosphatases, accelerating this pathologic process. Solid data support that elevated zinc may activate kinase GSK-3β, MAPKs and inhibit phosphatase PP-2A, thus promoting the hyperphosphorylation of tau and APP. Although the causal relationship between zinc disturbance and tau/Aβ pathology remains to be further clarified, targeting the zinc dyshomeostasis, thus preventing the disturbance of related kinases and phosphatases, may be a new approach for the intervention of these neurodegenerative diseases, which is worthy of further extensive exploration.

## Figures and Tables

**Table 1:** Zinc disruption in regulating protein kinases and protein phosphatases in neurodegenerative diseases.

Protein Kinase or Phosphatase	Expression Level or Activity in Disease Progression	Effect and Regulating Pathways of Zinc in Disease	Reference
GSK-3β	AD↑	Activation zinc-MEK/ERK/GSK-3β	[81,140]
CDK5	AD↑; ALS↑; PD ↑	Activation, To be identified	[141,142,143]
ERK	AD↑; PD↑	Activation zinc-Ras/Raf/MEK/ERK	[144,145,146,147]
JNK	AD↑; ALS↑	Activation To be identified	[148,149,150]
p38	AD↑	Activation To be identified	[147,148]
PKA	AD↑; PD↑	Not explored	[151,152]
PKC	AD↓	Not explored	[153,154]
PP1	AD↓	Not explored	[114]
PP2A	AD↓	Inhibition Direct effect, or through zinc-Src-PP2A(Y307)	[131,155]
PP2B	HD↓	Inhibition To be identified	[135]
PP5	AD↓	Not explored	[156]

AD, Alzheimer’s disease; ALS, Amyotrophic lateral sclerosis; CDK5, Cyclin-dependent kinase 5; ERK, Extracellular signal-regulated kinase; HD, Huntington’s disease; GSK-3β, Glycogen synthase kinase 3β; JNK, c-jun N-terminal kinase; PD, Parkinson’s disease; PKA, Protein kinase A; PKC, Protein kinase C; PP1, Protein phosphatase; PP2A, Protein phosphatase 2A; PP2B, Protein phosphatase 2B; PP5, Protein phosphatase 5.

## Data Availability

Not applicable.

## References

[1] O’Dell B.L., Newberne P.M., Savage J.E. (1958). Significance of dietary zinc for the growing chicken. J. Nutr..

[2] Sommer A.L., Lipman C.B. (1926). Evidence on the indispensable nature of zinc and boron for higher green plants. Plant Physiol..

[3] Jackson M. (1989). Physiology of zinc: General aspects. Zinc in Human Biology.

[4] Liuzzi J.P., Bobo J.A., Lichten L.A., Samuelson D.A., Cousins R.J. (2004). Responsive transporter genes within the murine intestinal-pancreatic axis form a basis of zinc homeostasis. Proc. Natl. Acad. Sci. USA.

[5] Vallee B.L., Falchuk K.H. (1993). The biochemical basis of zinc physiology. Physiol. Rev..

[6] Fukada T., Kambe T. (2011). Molecular and genetic features of zinc transporters in physiology and pathogenesis. Metallomics.

[7] Maret W., Li Y. (2009). Coordination dynamics of zinc in proteins. Chem. Rev..

[8] Devirgiliis C., Zalewski P.D., Perozzi G., Murgia C. (2007). Zinc fluxes and zinc transporter genes in chronic diseases. Mutat. Res..

[9] Fraker P.J., King L.E. (2004). Reprogramming of the immune system during zinc deficiency. Annu. Rev. Nutr..

[10] Prasad A.S. (1991). Discovery of human zinc deficiency and studies in an experimental human model. Am. J. Clin. Nutr..

[11] Frederickson C.J. (1989). Neurobiology of zinc and zinc-containing neurons. Int. Rev. Neurobiol..

[12] Ibata Y., Otsuka N. (1969). Electron microscopic demonstration of zinc in the hippocampal formation using Timm’s sulfide silver technique. J. Histochem. Cytochem..

[13] Coyle P., Philcox J.C., Carey L.C., Rofe A.M. (2002). Metallothionein: The multipurpose protein. Cell. Mol. Life Sci..

[14] Kambe T., Hashimoto A., Fujimoto S. (2014). Current understanding of ZIP and ZnT zinc transporters in human health and diseases. Cell. Mol. Life Sci..

[15] Lichten L.A., Cousins R.J. (2009). Mammalian zinc transporters: Nutritional and physiologic regulation. Annu. Rev. Nutr..

[16] Xu Y., Xiao G., Liu L., Lang M. (2019). Zinc transporters in Alzheimer’s disease. Mol. Brain.

[17] Bitanihirwe B.K., Cunningham M.G. (2009). Zinc: The brain’s dark horse. Synapse.

[18] Choi D.W., Koh J.Y. (1998). Zinc and brain injury. Annu. Rev. Neurosci..

[19] Minami A., Takeda A., Yamaide R., Oku N. (2002). Relationship between zinc and neurotransmitters released into the amygdalar extracellular space. Brain Res..

[20] Assaf S.Y., Chung S.H. (1984). Release of endogenous Zn^2+^ from brain tissue during activity. Nature.

[21] Vogt K., Mellor J., Tong G., Nicoll R. (2000). The actions of synaptically released zinc at hippocampal mossy fiber synapses. Neuron.

[22] Huiliang Z., Mengzhe Y., Xiaochuan W., Hui W., Min D., Mengqi W., Jianzhi W., Zhongshan C., Caixia P., Rong L. (2021). Zinc induces reactive astrogliosis through ERK-dependent activation of Stat3 and promotes synaptic degeneration. J. Neurochem..

[23] Kauppinen T.M., Higashi Y., Suh S.W., Escartin C., Nagasawa K., Swanson R.A. (2008). Zinc triggers microglial activation. J. Neurosci..

[24] Choi D.W., Yokoyama M., Koh J. (1988). Zinc neurotoxicity in cortical cell culture. Neuroscience.

[25] Sensi S.L., Yin H.Z., Carriedo S.G., Rao S.S., Weiss J.H. (1999). Preferential Zn^2+^ influx through Ca^2+^-permeable AMPA/kainate channels triggers prolonged mitochondrial superoxide production. Proc. Natl. Acad. Sci. USA.

[26] Plum L.M., Rink L., Haase H. (2010). The essential toxin: Impact of zinc on human health. Int. J. Environ. Res. Public Health.

[27] Yang Y., Jing X.P., Zhang S.P., Gu R.X., Tang F.X., Wang X.L., Xiong Y., Qiu M., Sun X.Y., Ke D. (2013). High dose zinc supplementation induces hippocampal zinc deficiency and memory impairment with inhibition of BDNF signaling. PLoS ONE.

[28] Barnham K.J., Bush A.I. (2014). Biological metals and metal-targeting compounds in major neurodegenerative diseases. Chem. Soc. Rev..

[29] Calap-Quintana P., González-Fernández J., Sebastiá-Ortega N., Llorens J.V., Moltó M.D. (2017). Drosophila melanogaster Models of Metal-Related Human Diseases and Metal Toxicity. Int. J. Mol. Sci..

[30] Shaw B.F., Valentine J.S. (2007). How do ALS-associated mutations in superoxide dismutase 1 promote aggregation of the protein?. Trends Biochem. Sci..

[31] Xiao G., Zhou B. (2016). What can flies tell us about zinc homeostasis?. Arch. Biochem. Biophys..

[32] Samii A., Nutt J.G., Ransom B.R. (2004). Parkinson’s disease. Lancet.

[33] Calabresi P., Di Filippo M., Gallina A., Wang Y., Stankowski J.N., Picconi B., Dawson V.L., Dawson T.M. (2013). New synaptic and molecular targets for neuroprotection in Parkinson’s disease. Mov. Disord..

[34] Granzotto A., Sensi S.L. (2015). Intracellular zinc is a critical intermediate in the excitotoxic cascade. Neurobiol. Dis..

[35] Surmeier D.J., Obeso J.A., Halliday G.M. (2017). Selective neuronal vulnerability in Parkinson disease. Nat. Rev. Neurosci..

[36] Tang J., Lu L., Wang Q., Liu H., Xue W., Zhou T., Xu L., Wang K., Wu D., Wei F. (2020). Crocin Reverses Depression-Like Behavior in Parkinson Disease Mice via VTA-mPFC Pathway. Mol. Neurobiol..

[37] Ahmed S.S., Santosh W. (2010). Metallomic profiling and linkage map analysis of early Parkinson’s disease: A new insight to aluminum marker for the possible diagnosis. PLoS ONE.

[38] Hegde M.L., Shanmugavelu P., Vengamma B., Rao T.S., Menon R.B., Rao R.V., Rao K.S. (2004). Serum trace element levels and the complexity of inter-element relations in patients with Parkinson’s disease. J. Trace Elem. Med. Biol..

[39] Alimonti A., Bocca B., Pino A., Ruggieri F., Forte G., Sancesario G. (2007). Elemental profile of cerebrospinal fluid in patients with Parkinson’s disease. J. Trace Elem. Med. Biol..

[40] Forte G., Bocca B., Senofonte O., Petrucci F., Brusa L., Stanzione P., Zannino S., Violante N., Alimonti A., Sancesario G. (2004). Trace and major elements in whole blood, serum, cerebrospinal fluid and urine of patients with Parkinson’s disease. J. Neural Transm..

[41] Ajjimaporn A., Phansuwan-Pujito P., Ebadi M., Govitrapong P. (2007). Zinc protects SK-N-SH cells from methamphetamine-induced alpha-synuclein expression. Neurosci. Lett..

[42] Ajjimaporn A., Shavali S., Ebadi M., Govitrapong P. (2008). Zinc rescues dopaminergic SK-N-SH cell lines from methamphetamine-induced toxicity. Brain Res. Bull..

[43] Saini N., Schaffner W. (2010). Zinc supplement greatly improves the condition of parkin mutant Drosophila. Biol. Chem..

[44] Pals P., Van Everbroeck B., Grubben B., Viaene M.K., Dom R., van der Linden C., Santens P., Martin J.J., Cras P. (2003). Case-control study of environmental risk factors for Parkinson’s disease in Belgium. Eur. J. Epidemiol..

[45] Dexter D.T., Carayon A., Javoy-Agid F., Agid Y., Wells F.R., Daniel S.E., Lees A.J., Jenner P., Marsden C.D. (1991). Alterations in the levels of iron, ferritin and other trace metals in Parkinson’s disease and other neurodegenerative diseases affecting the basal ganglia. Brain.

[46] Hussain S., Ali S.F. (2002). Zinc potentiates 1-methyl-4-phenyl-1,2,3,6-tetrahydropyridine induced dopamine depletion in caudate nucleus of mice brain. Neurosci. Lett..

[47] Bruijn L.I., Miller T.M., Cleveland D.W. (2004). Unraveling the mechanisms involved in motor neuron degeneration in ALS. Annu. Rev. Neurosci..

[48] Dang T.N., Lim N.K., Grubman A., Li Q.X., Volitakis I., White A.R., Crouch P.J. (2014). Increased metal content in the TDP-43(A315T) transgenic mouse model of frontotemporal lobar degeneration and amyotrophic lateral sclerosis. Front. Aging Neurosci..

[49] Sillevis Smitt P.A., Mulder T.P., Verspaget H.W., Blaauwgeers H.G., Troost D., de Jong J.M. (1994). Metallothionein in amyotrophic lateral sclerosis. Biol. Signals.

[50] Winblad B., Amouyel P., Andrieu S., Ballard C., Brayne C., Brodaty H., Cedazo-Minguez A., Dubois B., Edvardsson D., Feldman H. (2016). Defeating Alzheimer’s disease and other dementias: A priority for European science and society. Lancet. Neurology.

[51] Wortmann M. (2012). Dementia: A global health priority—Highlights from an ADI and World Health Organization report. Alzheimer’s Res. Ther..

[52] Lashley T., Schott J.M., Weston P., Murray C.E., Wellington H., Keshavan A., Foti S.C., Foiani M., Toombs J., Rohrer J.D. (2018). Molecular biomarkers of Alzheimer’s disease: Progress and prospects. Dis. Models Mech..

[53] Overk C.R., Masliah E. (2014). Pathogenesis of synaptic degeneration in Alzheimer’s disease and Lewy body disease. Biochem. Pharmacol..

[54] Danscher G., Jensen K.B., Frederickson C.J., Kemp K., Andreasen A., Juhl S., Stoltenberg M., Ravid R. (1997). Increased amount of zinc in the hippocampus and amygdala of Alzheimer’s diseased brains: A proton-induced X-ray emission spectroscopic analysis of cryostat sections from autopsy material. J. Neurosci. Methods.

[55] Deibel M.A., Ehmann W.D., Markesbery W.R. (1996). Copper, iron, and zinc imbalances in severely degenerated brain regions in Alzheimer’s disease: Possible relation to oxidative stress. J. Neurol. Sci..

[56] Religa D., Strozyk D., Cherny R.A., Volitakis I., Haroutunian V., Winblad B., Naslund J., Bush A.I. (2006). Elevated cortical zinc in Alzheimer disease. Neurology.

[57] Lovell M.A., Robertson J.D., Teesdale W.J., Campbell J.L., Markesbery W.R. (1998). Copper, iron and zinc in Alzheimer’s disease senile plaques. J. Neurol. Sci..

[58] Lovell M.A. (2009). A potential role for alterations of zinc and zinc transport proteins in the progression of Alzheimer’s disease. J. Alzheimer’s Dis..

[59] Lee M.C., Yu W.C., Shih Y.H., Chen C.Y., Guo Z.H., Huang S.J., Chan J.C.C., Chen Y.R. (2018). Zinc ion rapidly induces toxic, off-pathway amyloid-β oligomers distinct from amyloid-β derived diffusible ligands in Alzheimer’s disease. Sci. Rep..

[60] Sharma A.K., Pavlova S.T., Kim J., Kim J., Mirica L.M. (2013). The effect of Cu(2+) and Zn(2+) on the Aβ42 peptide aggregation and cellular toxicity. Metallomics.

[61] Bush A.I., Pettingell W.H., Multhaup G., d Paradis M., Vonsattel J.P., Gusella J.F., Beyreuther K., Masters C.L., Tanzi R.E. (1994). Rapid induction of Alzheimer A beta amyloid formation by zinc. Science.

[62] Adlard P.A., West A.K., Vickers J.C. (1998). Increased density of metallothionein I/II-immunopositive cortical glial cells in the early stages of Alzheimer’s disease. Neurobiol. Dis..

[63] Lovell M.A., Smith J.L., Xiong S., Markesbery W.R. (2005). Alterations in zinc transporter protein-1 (ZnT-1) in the brain of subjects with mild cognitive impairment, early, and late-stage Alzheimer’s disease. Neurotox. Res..

[64] Lyubartseva G., Smith J.L., Markesbery W.R., Lovell M.A. (2010). Alterations of zinc transporter proteins ZnT-1, ZnT-4 and ZnT-6 in preclinical Alzheimer’s disease brain. Brain Pathol..

[65] Whitfield D.R., Vallortigara J., Alghamdi A., Howlett D., Hortobágyi T., Johnson M., Attems J., Newhouse S., Ballard C., Thomas A.J. (2014). Assessment of ZnT3 and PSD95 protein levels in Lewy body dementias and Alzheimer’s disease: Association with cognitive impairment. Neurobiol. Aging.

[66] Bush A.I., Pettingell W.H., Paradis M.D., Tanzi R.E. (1994). Modulation of A beta adhesiveness and secretase site cleavage by zinc. J. Biol. Chem..

[67] Sun X.Y., Wei Y.P., Xiong Y., Wang X.C., Xie A.J., Wang X.L., Yang Y., Wang Q., Lu Y.M., Liu R. (2012). Synaptic released zinc promotes tau hyperphosphorylation by inhibition of protein phosphatase 2A (PP2A). J. Biol. Chem..

[68] Boom A., Authelet M., Dedecker R., Frédérick C., Van Heurck R., Daubie V., Leroy K., Pochet R., Brion J.P. (2009). Bimodal modulation of tau protein phosphorylation and conformation by extracellular Zn^2+^ in human-tau transfected cells. Biochim. Biophys. Acta.

[69] Kim I., Park E.J., Seo J., Ko S.J., Lee J., Kim C.H. (2011). Zinc stimulates tau S214 phosphorylation by the activation of Raf/mitogen-activated protein kinase-kinase/extracellular signal-regulated kinase pathway. Neuroreport.

[70] Frederickson C.J., Koh J.Y., Bush A.I. (2005). The neurobiology of zinc in health and disease. Nat. Rev. Neurosci..

[71] Sensi S.L., Granzotto A., Siotto M., Squitti R. (2018). Copper and Zinc Dysregulation in Alzheimer’s Disease. Trends Pharmacol. Sci..

[72] Sensi S.L., Paoletti P., Bush A.I., Sekler I. (2009). Zinc in the physiology and pathology of the CNS. Nat. Rev. Neurosci..

[73] Baudier J., Lee S.H., Cole R.D. (1987). Separation of the different microtubule-associated tau protein species from bovine brain and their mode II phosphorylation by Ca^2+^/phospholipid-dependent protein kinase C. J. Biol. Chem..

[74] Drewes G., Lichtenberg-Kraag B., Döring F., Mandelkow E.M., Biernat J., Goris J., Dorée M., Mandelkow E. (1992). Mitogen activated protein (MAP) kinase transforms tau protein into an Alzheimer-like state. EMBO J..

[75] Goedert M., Hasegawa M., Jakes R., Lawler S., Cuenda A., Cohen P. (1997). Phosphorylation of microtubule-associated protein tau by stress-activated protein kinases. FEBS Lett..

[76] Imahori K., Uchida T. (1997). Physiology and pathology of tau protein kinases in relation to Alzheimer’s disease. J. Biochem..

[77] Scott C.W., Spreen R.C., Herman J.L., Chow F.P., Davison M.D., Young J., Caputo C.B. (1993). Phosphorylation of recombinant tau by cAMP-dependent protein kinase. Identification of phosphorylation sites and effect on microtubule assembly. J. Biol. Chem..

[78] Embi N., Rylatt D.B., Cohen P. (1980). Glycogen synthase kinase-3 from rabbit skeletal muscle. Separation from cyclic-AMP-dependent protein kinase and phosphorylase kinase. Eur. J. Biochem..

[79] Pei J.J., Braak E., Braak H., Grundke-Iqbal I., Iqbal K., Winblad B., Cowburn R.F. (1999). Distribution of active glycogen synthase kinase 3beta (GSK-3beta) in brains staged for Alzheimer disease neurofibrillary changes. J. Neuropathol. Exp. Neurol..

[80] Pei J.J., Tanaka T., Tung Y.C., Braak E., Iqbal K., Grundke-Iqbal I. (1997). Distribution, levels, and activity of glycogen synthase kinase-3 in the Alzheimer disease brain. J. Neuropathol. Exp. Neurol..

[81] An W.L., Bjorkdahl C., Liu R., Cowburn R.F., Winblad B., Pei J.J. (2005). Mechanism of zinc-induced phosphorylation of p70 S6 kinase and glycogen synthase kinase 3beta in SH-SY5Y neuroblastoma cells. J. Neurochem..

[82] Frame S., Cohen P., Biondi R.M. (2001). A common phosphate binding site explains the unique substrate specificity of GSK3 and its inactivation by phosphorylation. Mol. Cell.

[83] Min Y.K., Lee J.E., Chung K.C. (2007). Zinc induces cell death in immortalized embryonic hippocampal cells via activation of Akt-GSK-3beta signaling. Exp. Cell Res..

[84] Ly P.T., Wu Y., Zou H., Wang R., Zhou W., Kinoshita A., Zhang M., Yang Y., Cai F., Woodgett J. (2013). Inhibition of GSK3β-mediated BACE1 expression reduces Alzheimer-associated phenotypes. J. Clin. Investig..

[85] Arioka M., Tsukamoto M., Ishiguro K., Kato R., Sato K., Imahori K., Uchida T. (1993). Tau protein kinase II is involved in the regulation of the normal phosphorylation state of tau protein. J. Neurochem..

[86] Lund E.T., McKenna R., Evans D.B., Sharma S.K., Mathews W.R. (2001). Characterization of the in vitro phosphorylation of human tau by tau protein kinase II (cdk5/p20) using mass spectrometry. J. Neurochem..

[87] Tuo Q.Z., Liuyang Z.Y., Lei P., Yan X., Shentu Y.P., Liang J.W., Zhou H., Pei L., Xiong Y., Hou T.Y. (2018). Zinc induces CDK5 activation and neuronal death through CDK5-Tyr15 phosphorylation in ischemic stroke. Cell Death Dis..

[88] Guo C., Wang P., Zhong M.L., Wang T., Huang X.S., Li J.Y., Wang Z.Y. (2013). Deferoxamine inhibits iron induced hippocampal tau phosphorylation in the Alzheimer transgenic mouse brain. Neurochem. Int..

[89] Iijima K., Ando K., Takeda S., Satoh Y., Seki T., Itohara S., Greengard P., Kirino Y., Nairn A.C., Suzuki T. (2000). Neuron-specific phosphorylation of Alzheimer’s beta-amyloid precursor protein by cyclin-dependent kinase 5. J. Neurochem..

[90] Johnson G.L., Lapadat R. (2002). Mitogen-activated protein kinase pathways mediated by ERK, JNK, and p38 protein kinases. Science.

[91] Kim E.K., Choi E.J. (2015). Compromised MAPK signaling in human diseases: An update. Arch. Toxicol..

[92] McLaughlin B., Pal S., Tran M.P., Parsons A.A., Barone F.C., Erhardt J.A., Aizenman E. (2001). p38 activation is required upstream of potassium current enhancement and caspase cleavage in thiol oxidant-induced neuronal apoptosis. J. Neurosci..

[93] Seo S.R., Chong S.A., Lee S.I., Sung J.Y., Ahn Y.S., Chung K.C., Seo J.T. (2001). Zn^2+^-induced ERK activation mediated by reactive oxygen species causes cell death in differentiated PC12 cells. J. Neurochem..

[94] Colombo A., Bastone A., Ploia C., Sclip A., Salmona M., Forloni G., Borsello T. (2009). JNK regulates APP cleavage and degradation in a model of Alzheimer’s disease. Neurobiol. Dis..

[95] Zhou Q., Wang M., Du Y., Zhang W., Bai M., Zhang Z., Li Z., Miao J. (2015). Inhibition of c-Jun N-terminal kinase activation reverses Alzheimer disease phenotypes in APPswe/PS1dE9 mice. Ann. Neurol..

[96] Lee J.K., Kim N.J. (2017). Recent Advances in the Inhibition of p38 MAPK as a Potential Strategy for the Treatment of Alzheimer’s Disease. Molecules.

[97] Siano G., Caiazza M.C., Ollà I., Varisco M., Madaro G., Quercioli V., Calvello M., Cattaneo A., Di Primio C. (2019). Identification of an ERK Inhibitor as a Therapeutic Drug Against Tau Aggregation in a New Cell-Based Assay. Front. Cell. Neurosci..

[98] Carlyle B.C., Nairn A.C., Wang M., Yang Y., Jin L.E., Simen A.A., Ramos B.P., Bordner K.A., Craft G.E., Davies P. (2014). cAMP-PKA phosphorylation of tau confers risk for degeneration in aging association cortex. Proc. Natl. Acad. Sci. USA.

[99] Paspalas C.D., Carlyle B.C., Leslie S., Preuss T.M., Crimins J.L., Huttner A.J., van Dyck C.H., Rosene D.L., Nairn A.C., Arnsten A.F.T. (2018). The aged rhesus macaque manifests Braak stage III/IV Alzheimer’s-like pathology. Alzheimer’s Dement..

[100] Allouche-Fitoussi D., Breitbart H. (2020). The Role of Zinc in Male Fertility. Int. J. Mol. Sci..

[101] Park K.H., Choi Y., Yoon D.S., Lee K.M., Kim D., Lee J.W. (2018). Zinc Promotes Osteoblast Differentiation in Human Mesenchymal Stem Cells Via Activation of the cAMP-PKA-CREB Signaling Pathway. Stem Cells Dev..

[102] Kang J.H., Toita R., Kim C.W., Katayama Y. (2012). Protein kinase C (PKC) isozyme-specific substrates and their design. Biotechnol. Adv..

[103] Mochly-Rosen D., Das K., Grimes K.V. (2012). Protein kinase C, an elusive therapeutic target?. Nat. Rev. Drug Discov..

[104] Steinberg S.F. (2008). Structural basis of protein kinase C isoform function. Physiol. Rev..

[105] Isagawa T., Mukai H., Oishi K., Taniguchi T., Hasegawa H., Kawamata T., Tanaka C., Ono Y. (2000). Dual effects of PKNalpha and protein kinase C on phosphorylation of tau protein by glycogen synthase kinase-3beta. Biochem. Biophys. Res. Commun..

[106] Takashima A. (2006). GSK-3 is essential in the pathogenesis of Alzheimer’s disease. J. Alzheimer’s Dis..

[107] Correas I., Díaz-Nido J., Avila J. (1992). Microtubule-associated protein tau is phosphorylated by protein kinase C on its tubulin binding domain. J. Biol. Chem..

[108] Noh K.M., Koh J.Y. (2000). Induction and activation by zinc of NADPH oxidase in cultured cortical neurons and astrocytes. J. Neurosci..

[109] Noh K.M., Kim Y.H., Koh J.Y. (1999). Mediation by membrane protein kinase C of zinc-induced oxidative neuronal injury in mouse cortical cultures. J. Neurochem..

[110] Sajan M.P., Hansen B.C., Higgs M.G., Kahn C.R., Braun U., Leitges M., Park C.R., Diamond D.M., Farese R.V. (2018). Atypical PKC, PKCλ/ι, activates β-secretase and increases Aβ(1-40/42) and phospho-tau in mouse brain and isolated neuronal cells, and may link hyperinsulinemia and other aPKC activators to development of pathological and memory abnormalities in Alzheimer’s disease. Neurobiol. Aging.

[111] Olsen J.V., Blagoev B., Gnad F., Macek B., Kumar C., Mortensen P., Mann M. (2006). Global, in vivo, and site-specific phosphorylation dynamics in signaling networks. Cell.

[112] Kamada R., Kudoh F., Ito S., Tani I., Janairo J.I.B., Omichinski J.G., Sakaguchi K. (2020). Metal-dependent Ser/Thr protein phosphatase PPM family: Evolution, structures, diseases and inhibitors. Pharmacol. Ther..

[113] Shi Y. (2009). Serine/threonine phosphatases: Mechanism through structure. Cell.

[114] Gong C.X., Singh T.J., Grundke-Iqbal I., Iqbal K. (1993). Phosphoprotein phosphatase activities in Alzheimer disease brain. J. Neurochem..

[115] Liu F., Grundke-Iqbal I., Iqbal K., Gong C.X. (2005). Contributions of protein phosphatases PP1, PP2A, PP2B and PP5 to the regulation of tau phosphorylation. Eur. J. Neurosci..

[116] Rahman A., Grundke-Iqbal I., Iqbal K. (2005). Phosphothreonine-212 of Alzheimer abnormally hyperphosphorylated tau is a preferred substrate of protein phosphatase-1. Neurochem. Res..

[117] Kelker M.S., Page R., Peti W. (2009). Crystal structures of protein phosphatase-1 bound to nodularin-R and tautomycin: A novel scaffold for structure-based drug design of serine/threonine phosphatase inhibitors. J. Mol. Biol..

[118] Zhang A.J., Bai G., Deans-Zirattu S., Browner M.F., Lee E.Y. (1992). Expression of the catalytic subunit of phosphorylase phosphatase (protein phosphatase-1) in *Escherichia coli*. J. Biol. Chem..

[119] Chu Y., Lee E.Y., Schlender K.K. (1996). Activation of protein phosphatase 1. Formation of a metalloenzyme. J. Biol. Chem..

[120] Singla N., Dhawan D.K. (2012). Regulatory role of zinc during aluminium-induced altered carbohydrate metabolism in rat brain. J. Neurosci. Res..

[121] Gong C.X., Shaikh S., Wang J.Z., Zaidi T., Grundke-Iqbal I., Iqbal K. (1995). Phosphatase activity toward abnormally phosphorylated tau: Decrease in Alzheimer disease brain. J. Neurochem..

[122] Sun L., Liu S.Y., Zhou X.W., Wang X.C., Liu R., Wang Q., Wang J.Z. (2003). Inhibition of protein phosphatase 2A- and protein phosphatase 1-induced tau hyperphosphorylation and impairment of spatial memory retention in rats. Neuroscience.

[123] Sontag E., Nunbhakdi-Craig V., Sontag J.M., Diaz-Arrastia R., Ogris E., Dayal S., Lentz S.R., Arning E., Bottiglieri T. (2007). Protein phosphatase 2A methyltransferase links homocysteine metabolism with tau and amyloid precursor protein regulation. J. Neurosci..

[124] Ingebritsen T.S., Cohen P. (1983). The protein phosphatases involved in cellular regulation. 1. Classification and substrate specificities. Eur. J. Biochem..

[125] Sangodkar J., Farrington C.C., McClinch K., Galsky M.D., Kastrinsky D.B., Narla G. (2016). All roads lead to PP2A: Exploiting the therapeutic potential of this phosphatase. FEBS J..

[126] Nishito Y., Usui H., Tanabe O., Shimizu M., Takeda M. (1999). Interconversion of Mn(2+)-dependent and -independent protein phosphatase 2A from human erythrocytes: Role of Zn(2+) and Fe(2+) in protein phosphatase 2A. J. Biochem..

[127] Cohen P. (1989). The structure and regulation of protein phosphatases. Annu. Rev. Biochem..

[128] Janssens V., Goris J. (2001). Protein phosphatase 2A: A highly regulated family of serine/threonine phosphatases implicated in cell growth and signalling. Biochem. J..

[129] Xiong Y., Luo D.J., Wang X.L., Qiu M., Yang Y., Yan X., Wang J.Z., Ye Q.F., Liu R. (2015). Zinc binds to and directly inhibits protein phosphatase 2A in vitro. Neurosci. Bull..

[130] Chen J., Martin B.L., Brautigan D.L. (1992). Regulation of protein serine-threonine phosphatase type-2A by tyrosine phosphorylation. Science.

[131] Xiong Y., Jing X.P., Zhou X.W., Wang X.L., Yang Y., Sun X.Y., Qiu M., Cao F.Y., Lu Y.M., Liu R. (2013). Zinc induces protein phosphatase 2A inactivation and tau hyperphosphorylation through Src dependent PP2A (tyrosine 307) phosphorylation. Neurobiol. Aging.

[132] Ferreira A., Kincaid R., Kosik K.S. (1993). Calcineurin is associated with the cytoskeleton of cultured neurons and has a role in the acquisition of polarity. Mol. Biol. Cell.

[133] Gong C.X., Singh T.J., Grundke-Iqbal I., Iqbal K. (1994). Alzheimer’s disease abnormally phosphorylated tau is dephosphorylated by protein phosphatase-2B (calcineurin). J. Neurochem..

[134] Wei Q., Holzer M., Brueckner M.K., Liu Y., Arendt T. (2002). Dephosphorylation of tau protein by calcineurin triturated into neural living cells. Cell. Mol. Neurobiol..

[135] Gratuze M., Noël A., Julien C., Cisbani G., Milot-Rousseau P., Morin F., Dickler M., Goupil C., Bezeau F., Poitras I. (2015). Tau hyperphosphorylation and deregulation of calcineurin in mouse models of Huntington’s disease. Hum. Mol. Genet..

[136] Takahashi K., Akaishi E., Abe Y., Ishikawa R., Tanaka S., Hosaka K., Kubohara Y. (2003). Zinc inhibits calcineurin activity in vitro by competing with nickel. Biochem. Biophys. Res. Commun..

[137] Merten K.E., Jiang Y., Kang Y.J. (2007). Zinc inhibits doxorubicin-activated calcineurin signal transduction pathway in H9c2 embryonic rat cardiac cells. Exp. Biol. Med..

[138] Park K.H., Park B., Yoon D.S., Kwon S.H., Shin D.M., Lee J.W., Lee H.G., Shim J.H., Park J.H., Lee J.M. (2013). Zinc inhibits osteoclast differentiation by suppression of Ca2+-Calcineurin-NFATc1 signaling pathway. Cell Commun. Signal..

[139] Gong C.X., Liu F., Wu G., Rossie S., Wegiel J., Li L., Grundke-Iqbal I., Iqbal K. (2004). Dephosphorylation of microtubule-associated protein tau by protein phosphatase 5. J. Neurochem..

[140] Leroy K., Yilmaz Z., Brion J.P. (2007). Increased level of active GSK-3beta in Alzheimer’s disease and accumulation in argyrophilic grains and in neurones at different stages of neurofibrillary degeneration. Neuropathol. Appl. Neurobiol..

[141] Lee K.Y., Clark A.W., Rosales J.L., Chapman K., Fung T., Johnston R.N. (1999). Elevated neuronal Cdc2-like kinase activity in the Alzheimer disease brain. Neurosci. Res..

[142] Nguyen M.D., Larivière R.C., Julien J.P. (2001). Deregulation of Cdk5 in a mouse model of ALS: Toxicity alleviated by perikaryal neurofilament inclusions. Neuron.

[143] Smith P.D., Crocker S.J., Jackson-Lewis V., Jordan-Sciutto K.L., Hayley S., Mount M.P., O’Hare M.J., Callaghan S., Slack R.S., Przedborski S. (2003). Cyclin-dependent kinase 5 is a mediator of dopaminergic neuron loss in a mouse model of Parkinson’s disease. Proc. Natl. Acad. Sci. USA.

[144] Ferrer I., Blanco R., Carmona M., Ribera R., Goutan E., Puig B., Rey M.J., Cardozo A., Viñals F., Ribalta T. (2001). Phosphorylated map kinase (ERK1, ERK2) expression is associated with early tau deposition in neurones and glial cells, but not with increased nuclear DNA vulnerability and cell death, in Alzheimer disease, Pick’s disease, progressive supranuclear palsy and corticobasal degeneration. Brain Pathol..

[145] Kulich S.M., Chu C.T. (2001). Sustained extracellular signal-regulated kinase activation by 6-hydroxydopamine: Implications for Parkinson’s disease. J. Neurochem..

[146] Perry G., Roder H., Nunomura A., Takeda A., Friedlich A.L., Zhu X., Raina A.K., Holbrook N., Siedlak S.L., Harris P.L. (1999). Activation of neuronal extracellular receptor kinase (ERK) in Alzheimer disease links oxidative stress to abnormal phosphorylation. Neuroreport.

[147] Zhu X., Castellani R.J., Takeda A., Nunomura A., Atwood C.S., Perry G., Smith M.A. (2001). Differential activation of neuronal ERK, JNK/SAPK and p38 in Alzheimer disease: The ‘two hit’ hypothesis. Mech. Ageing Dev..

[148] Atzori C., Ghetti B., Piva R., Srinivasan A.N., Zolo P., Delisle M.B., Mirra S.S., Migheli A. (2001). Activation of the JNK/p38 pathway occurs in diseases characterized by tau protein pathology and is related to tau phosphorylation but not to apoptosis. J. Neuropathol. Exp. Neurol..

[149] Migheli A., Piva R., Atzori C., Troost D., Schiffer D. (1997). c-Jun, JNK/SAPK kinases and transcription factor NF-kappa B are selectively activated in astrocytes, but not motor neurons, in amyotrophic lateral sclerosis. J. Neuropathol. Exp. Neurol..

[150] Zhu X., Raina A.K., Rottkamp C.A., Aliev G., Perry G., Boux H., Smith M.A. (2001). Activation and redistribution of c-jun N-terminal kinase/stress activated protein kinase in degenerating neurons in Alzheimer’s disease. J. Neurochem..

[151] Dagda R.K., Das Banerjee T. (2015). Role of protein kinase A in regulating mitochondrial function and neuronal development: Implications to neurodegenerative diseases. Rev. Neurosci..

[152] Howells D.W., Porritt M.J., Wong J.Y., Batchelor P.E., Kalnins R., Hughes A.J., Donnan G.A. (2000). Reduced BDNF mRNA expression in the Parkinson’s disease substantia nigra. Exp. Neurol..

[153] Govoni S., Bergamaschi S., Racchi M., Battaini F., Binetti G., Bianchetti A., Trabucchi M. (1993). Cytosol protein kinase C downregulation in fibroblasts from Alzheimer’s disease patients. Neurology.

[154] Wang H.Y., Pisano M.R., Friedman E. (1994). Attenuated protein kinase C activity and translocation in Alzheimer’s disease brain. Neurobiol. Aging.

[155] Pei J.J., Gong C.X., Iqbal K., Grundke-Iqbal I., Wu Q.L., Winblad B., Cowburn R.F. (1998). Subcellular distribution of protein phosphatases and abnormally phosphorylated tau in the temporal cortex from Alzheimer’s disease and control brains. J. Neural Transm..

[156] Liu F., Iqbal K., Grundke-Iqbal I., Rossie S., Gong C.X. (2005). Dephosphorylation of tau by protein phosphatase 5: Impairment in Alzheimer’s disease. J. Biol. Chem..

